# Supplementary material for: Precise Slow Oscillation–Spindle Coupling Promotes Memory Consolidation in Younger and Older Adults
Source: Sci Rep. 2019 Feb 13;9:1940. doi: 10.1038/s41598-018-36557-z (PMC6374430; doi:10.1038/s41598-018-36557-z)
Supplement: Supplementary file 1 — Supplementary Information [file 41598_2018_36557_MOESM1_ESM.pdf]

SUPPLEMENTARY INFORMATION

Precise Slow Oscillation–Spindle Coupling Promotes Memory Consolidation in Younger and Older Adults

Beate E. Muehlroth[1*], Myriam C. Sander[1], Yana Fandakova[1], Thomas H. Grandy[1], Björn Rasch[2], Yee Lee Shing[1,3] and Markus Werkle-Bergner[1*]

[1]*Center for Lifespan Psychology, Max Planck Institute for Human Development, Berlin, Germany*

[2]*Department of Psychology, University of Fribourg, Fribourg, Switzerland*

[3]*Department of Developmental Psychology, Goethe University Frankfurt, Frankfurt am Main, Germany*

Correspondence concerning this article should be addressed to:

Beate E. Muehlroth (muehlroth@mpib-berlin.mpg.de) or Markus Werkle-Bergner (werkle@mpib-berlin.mpg.de);

Lentzeallee 94, 14195 Berlin, Germany

Supplementary Table S1. Age comparison of sleep oscillations

	YA – Median [1st quartile; 3rd quartile]	OA – Median [1st quartile; 3rd quartile]	*z*	*p*
**Slow oscillations** (frontal)				
number	1162.5 [1055.25; 1355.38]	970.5 [788.0; 1169.25]	3.14	.002
density (events/min)	3.83 [3.33; 4.18]	3.56 [2.89; 3.94]	2.09	.037
frequency (Hz)	0.79 [0.78; 0.81]	0.78 [0.75; 0.8]	2.68	.007
amplitude (μV)	123.54 [105.08; 140.11]	74.82 [60.45; 86.48]	5.69	<.001
**Slow spindles** (frontal)				
number	275.75 [194.25; 378.00]	141.50 [86.25; 228.50]	3.41	<.001
density (events/min)	0.83 [0.63; 1.08]	0.48 [0.32; 0.69]	3.16	.002
frequency (Hz)	11.04 [10.69; 11.36]	10.80 [10.64; 11.08]	1.39	.164
amplitude (μV)	23.69 [18.38; 27]	19.91 [16.21; 24.07]	1.94	.05
**Fast spindles** (Cz)				
number	511.0 [387.0; 654.5]	162.0 [130.0; 280.0]	5.89	<.001
density (events/min)	1.70 [1.35; 1.79]	0.58 [0.38; 0.97]	5.32	<.001
frequency (Hz)	13.45 [13.23; 13.68]	13.76 [13.56; 13.9]	–2.69	.007
amplitude (μV)	15.75 [13.33; 20.44]	13.65 [10.85; 15.35]	2.88	.004

Note. z and *p*-values were derived from non-parametric Mann-Whitney *U* tests comparing sleep parameters between younger and older adults. YA: younger adults; OA: older adults.

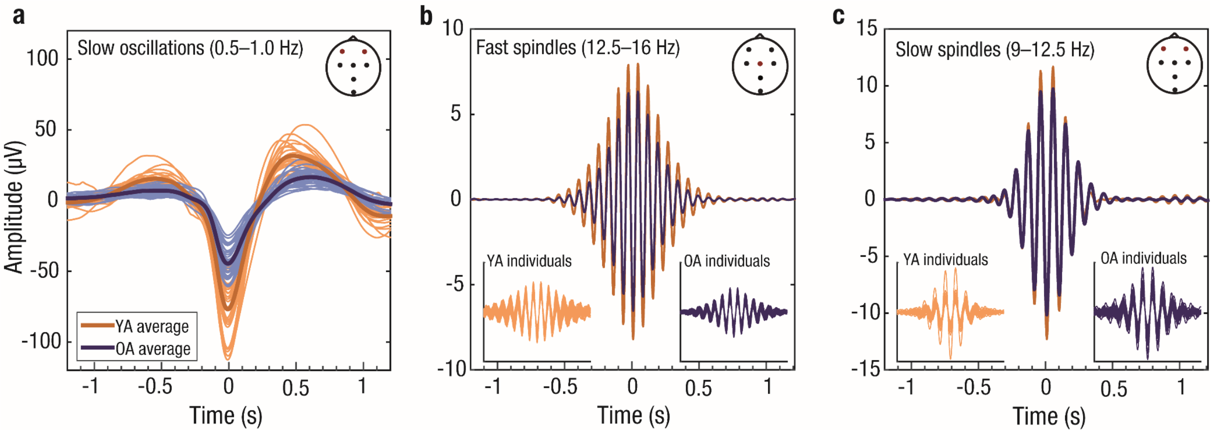

Supplementary Figure S1. Detected sleep oscillations in younger and older adults. Average events within the respective bandpass-filtered signal are depicted. (a) Average frontal SOs for younger (light orange) and older (light blue) individuals. Averages for each age group are plotted in thick dark lines. (b and c) Average and individual fast and slow SPs. Detected spindle events reveal the prominent waxing and waning shape of SPs. Panel (a) is the same as shown in Figure 4a and added here for completeness. SO: slow oscillation; SP: spindle.

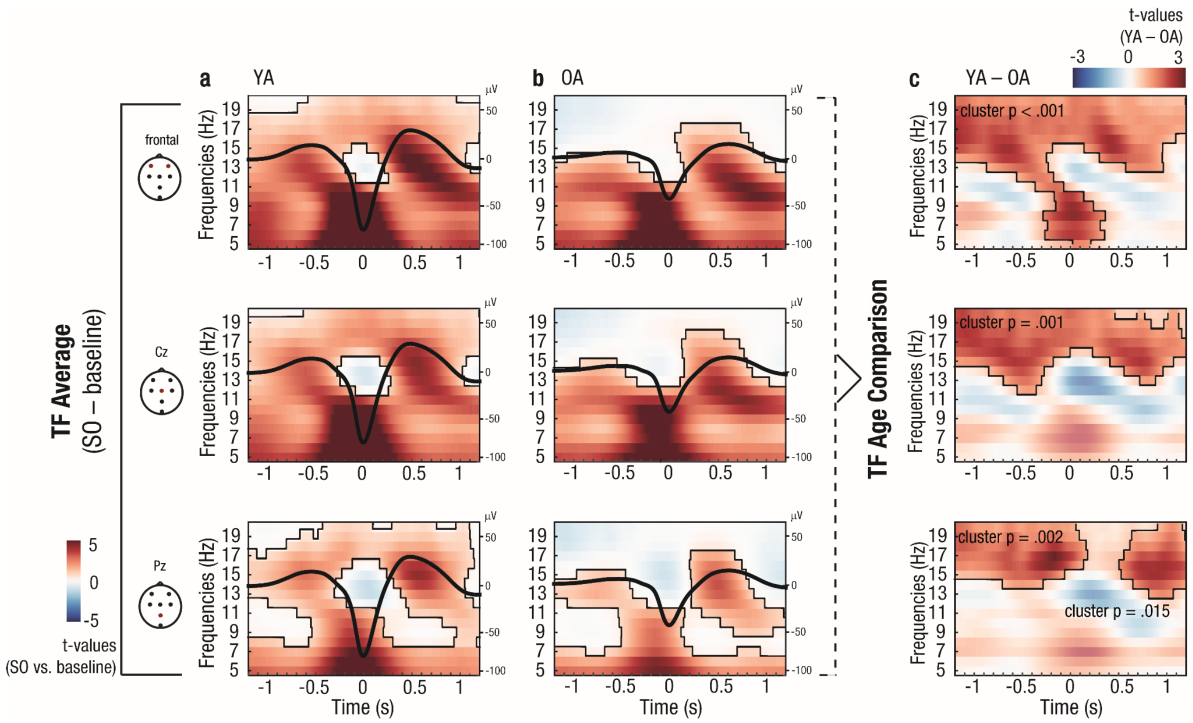

Supplementary Figure S2. The timing and topography of fast and slow SP activity during SOs differs for younger and older adults. (a and b) Differences in wavelet power for SO trials (respective trough ± 1.2 s) compared to baseline trials without SOs are depicted (in *t*-score units). Significant clusters (cluster-based permutation test, cluster α < 0.05) are highlighted and outlined. The average frontal SO for each age group is inserted in black (the scale in μV is indicated on the right of each time–frequency graph). In both age groups, EEG power is modulated as a function of the SO phase. (a) In younger adults, fast SP activity (12.5–16 Hz) peaks during the up-state in all derivations but the effect is most specific in centroparietal electrodes where it appears precisely during the peak of the SO. Slow SP power (9–12.5 Hz) is strongest at the up- to down-state transition in frontal channels and at Cz. (b) In older adults, a specific fast SP power peak is only seen at Pz but is less tied to the SO peak. In general, power increases are shifted to lower frequencies as compared to younger adults. (c) The *t*-map of the time–frequency age comparison is displayed (significant clusters highlighted and outlined, cluster α < 0.05). In all channels, significant age-related reductions in fast SP power are tied to the SO up-states only. YA: younger adults; OA: older adults; SO: slow oscillation; SP: spindle; TF: time–frequency.

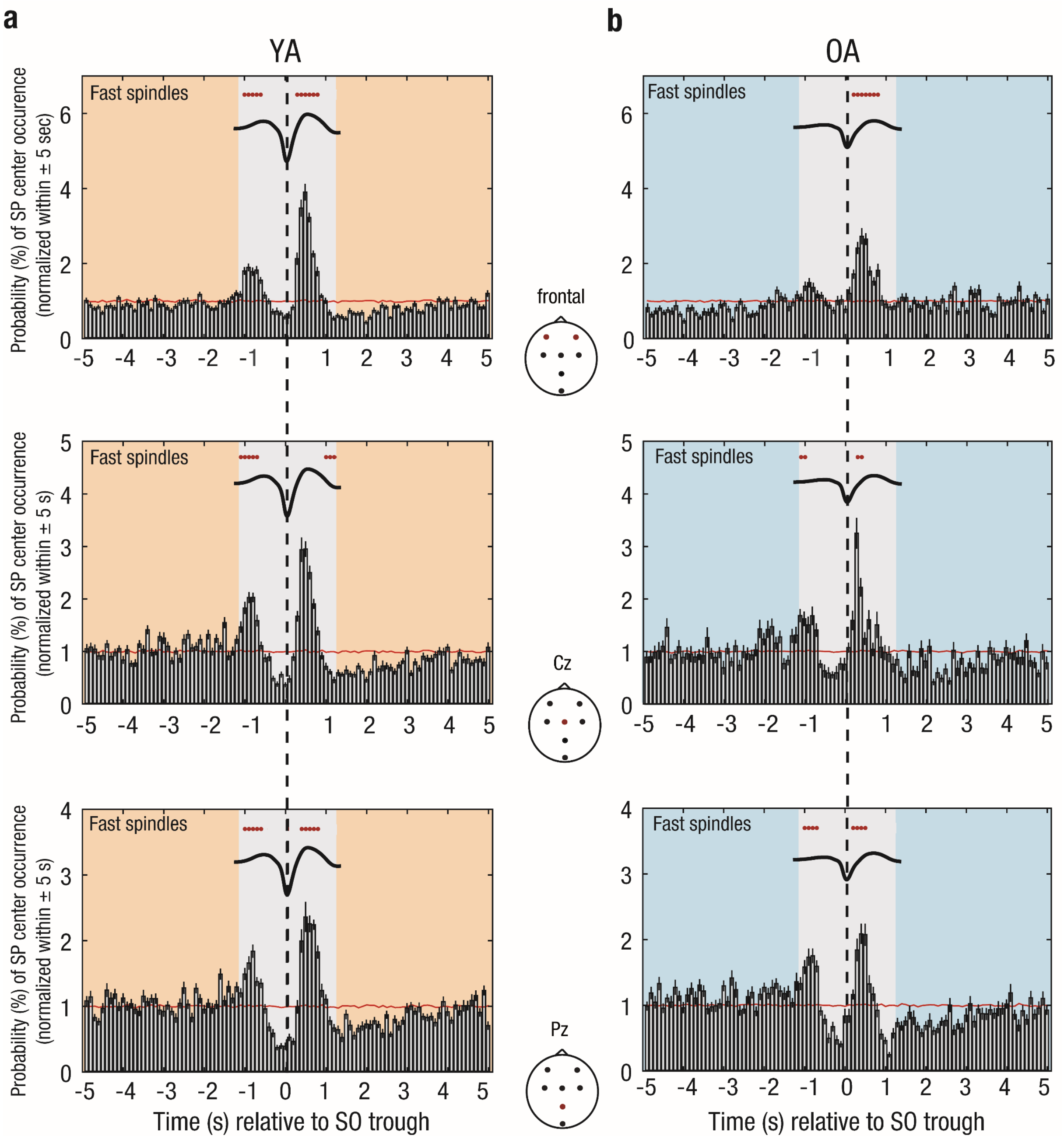

Supplementary Figure S3. The timing and topography of fast SP events differs for younger and older adults. Peri-event time histograms (PETHs) of fast SPs at frontal channels, Cz and Pz, co-occurring with frontal SOs, are shown for both age groups. PETHs are computed for time intervals of ± 5 s relative to the SO trough. Standard errors of the 100-ms bins are included as black vertical lines. The average SO for each age group is inserted and the corresponding time interval (± 1.2 s) is shaded in gray. Dashed vertical lines mark the trough of the SO. Reference distribution obtained after randomization of the data is shown in red. Red asterisks indicate significantly increased SP occurrence contrasted with the reference distribution (cluster-based permutation test, cluster α < 0.05, positive clusters only). (a) In younger adults, a distinct increase in fast SPs during the SO up-state is observed in all derivations. Significant fast SP increases are restricted to the SO interval only. (b) In older adults, similar to younger adults, significant fast SP increases are restricted to the SO interval only. The fast SP peak occurs before the SO peak, though. Overall, fast SP modulation is less specific, in particular at electrode Cz. YA: younger adults; OA: older adults; SO: slow oscillation; SP: spindle.

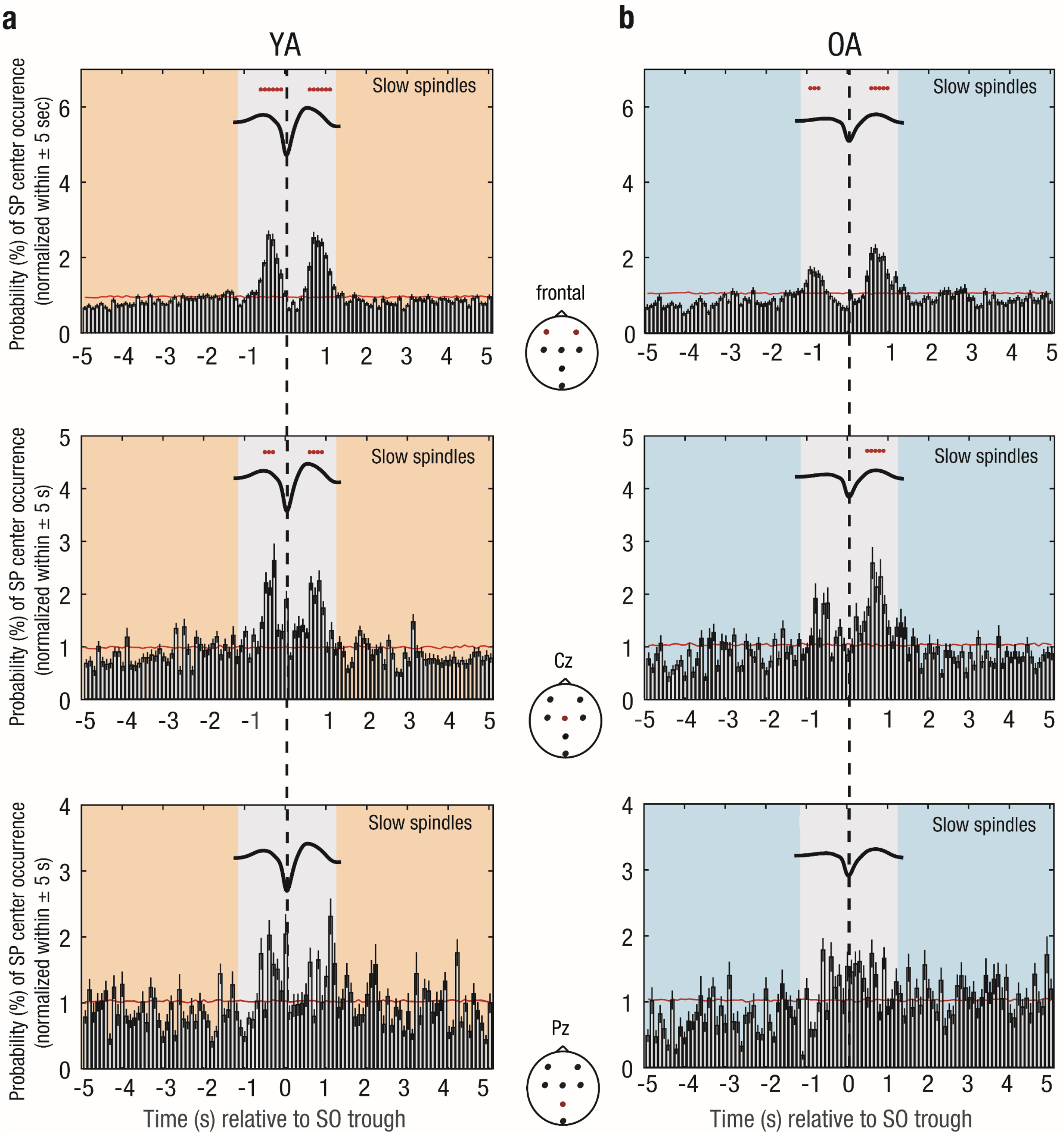

Supplementary Figure S4. Fine-tuned slow SP modulation is specific to frontal channels in both younger and older adults. Peri-event time histograms (PETHs) of slow SPs at frontal channels, Cz and Pz, co-occurring with frontal SOs, are shown for both age groups. PETHs are computed for time intervals of ± 5 s relative to the SO trough. Standard errors of the 100-ms bins are included as black vertical lines. The average SO for each age group is inserted and the corresponding time interval (± 1.2 s) is shaded in gray. Dashed vertical lines mark the trough of the SO. Reference distribution obtained after randomization of the data is shown in red. Red asterisks indicate significantly increased SP occurrence contrasted with the reference distribution (cluster-based permutation test, cluster $\alpha < 0.05$, positive clusters only). (a) In younger adults, slow SPs globally peak at the up- to down-state transition over frontal and central channels only. (b) In older adults, the frontal slow SP peak at the up- to down-state transition preceding the SO trough disappears. YA: younger adults; OA: older adults; SO: slow oscillation; SP: spindle.

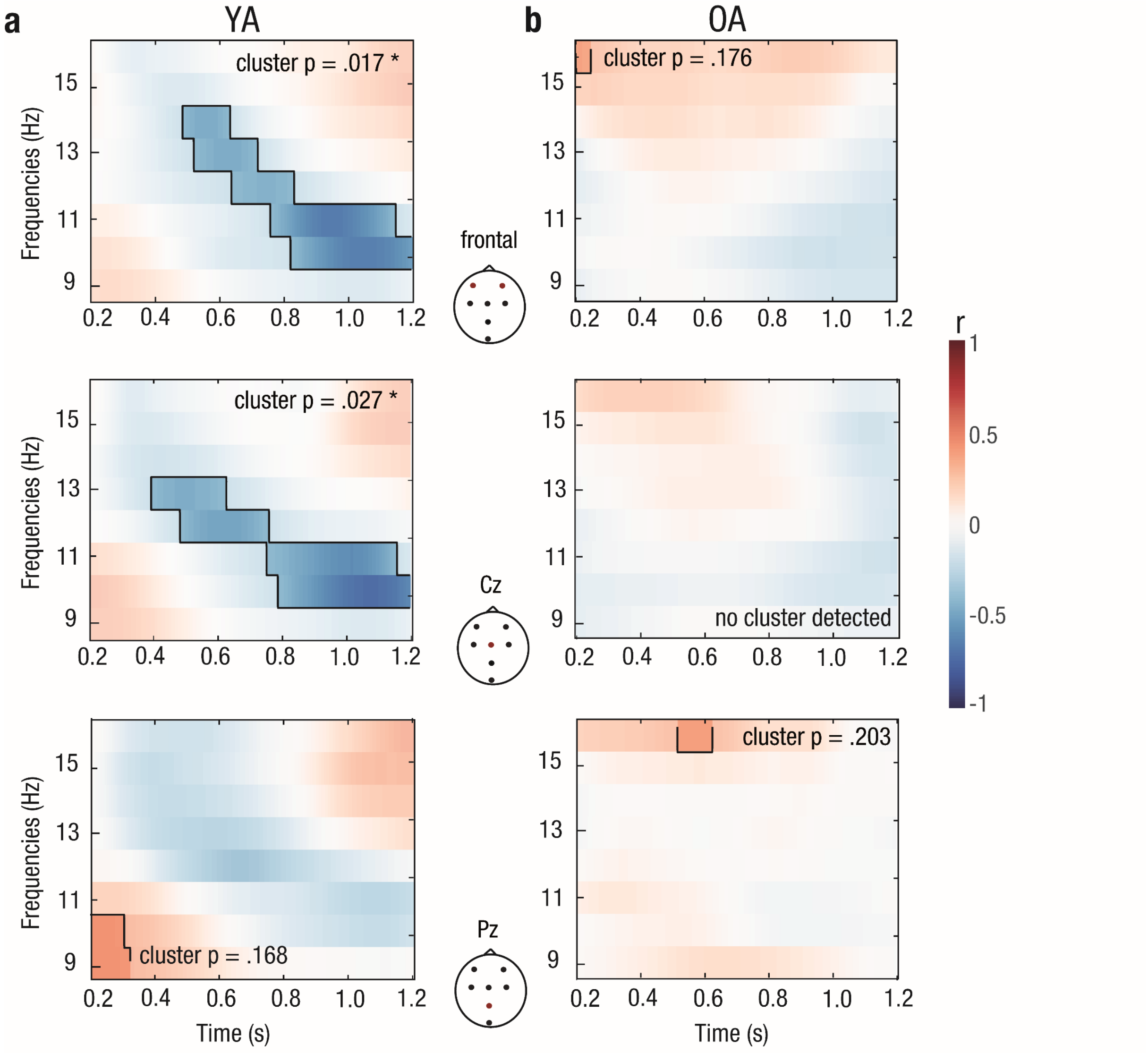

Supplementary Figure S5. Electrophysiological activity during the SO up-state predicts memory retention. Pearson's correlation coefficients between memory retention and neuronal activity during SO up-states are displayed for both age groups. In each plot the cluster with the smallest *p*-value (after control for multiple comparisons using a cluster-corrected correlation approach) is highlighted and outlined and its significance (cluster $\alpha < 0.05$) is marked by asterisks. (a) In younger adults, a significant negative correlation cluster appears over frontal channels and at Cz. More positive values in this cluster are associated with worse memory retention. (b) No robust cluster was detected in older adults. YA: younger adults; OA: older adults; SO: slow oscillation.

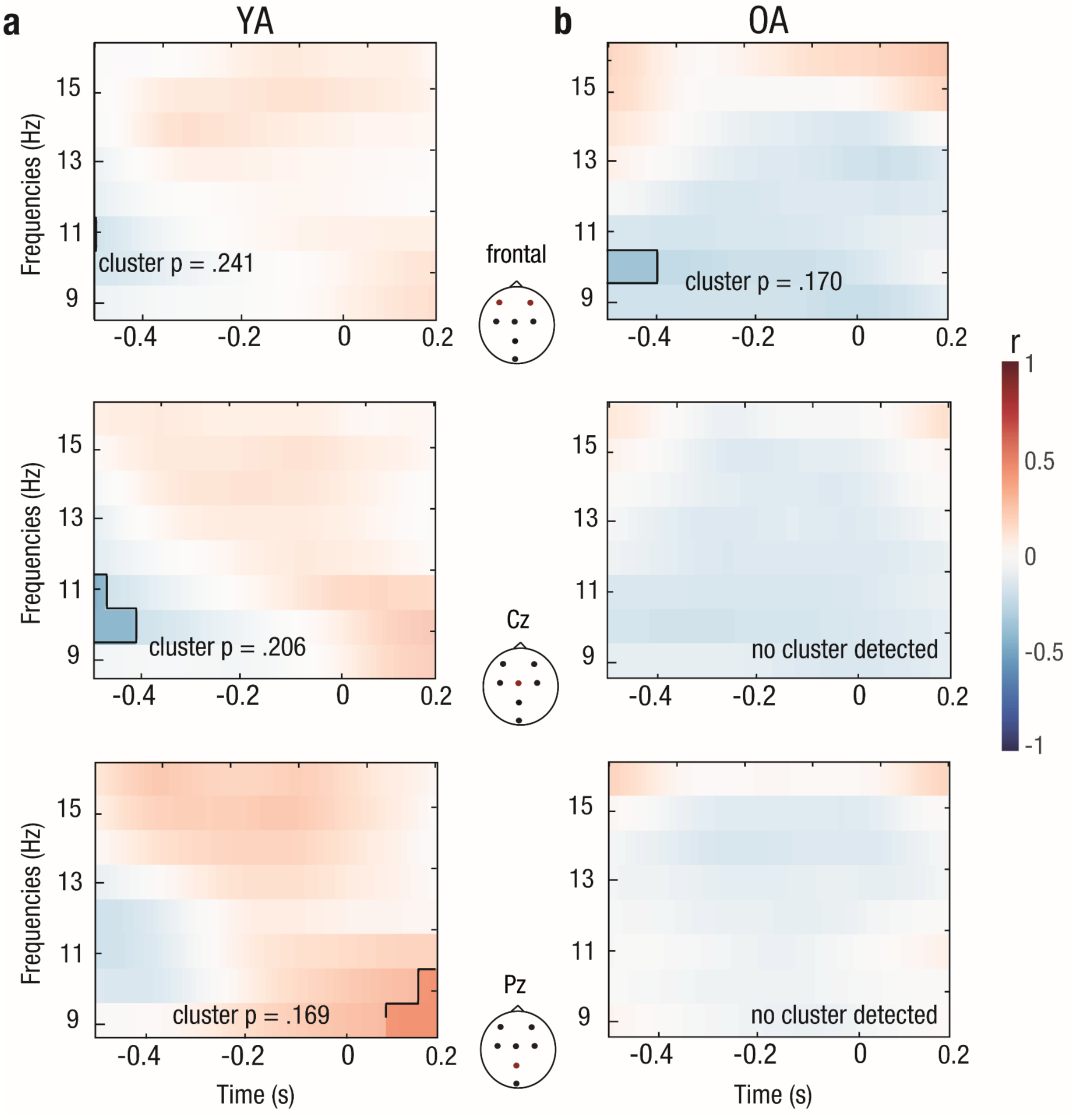

Supplementary Figure S6. Electrophysiological activity during the SO up- to down-state transition does not predict memory retention. Pearson's correlation coefficients between memory retention and neuronal activity at the up- to down-state transition are displayed for both age groups. In each plot the cluster with the smallest *p*-value (after control for multiple comparisons using a cluster-corrected correlation approach) is highlighted and outlined. (a and b) No robust association with memory retention was detected in neither younger nor in older adults. YA: younger adults; OA: older adults; SO: slow oscillation.

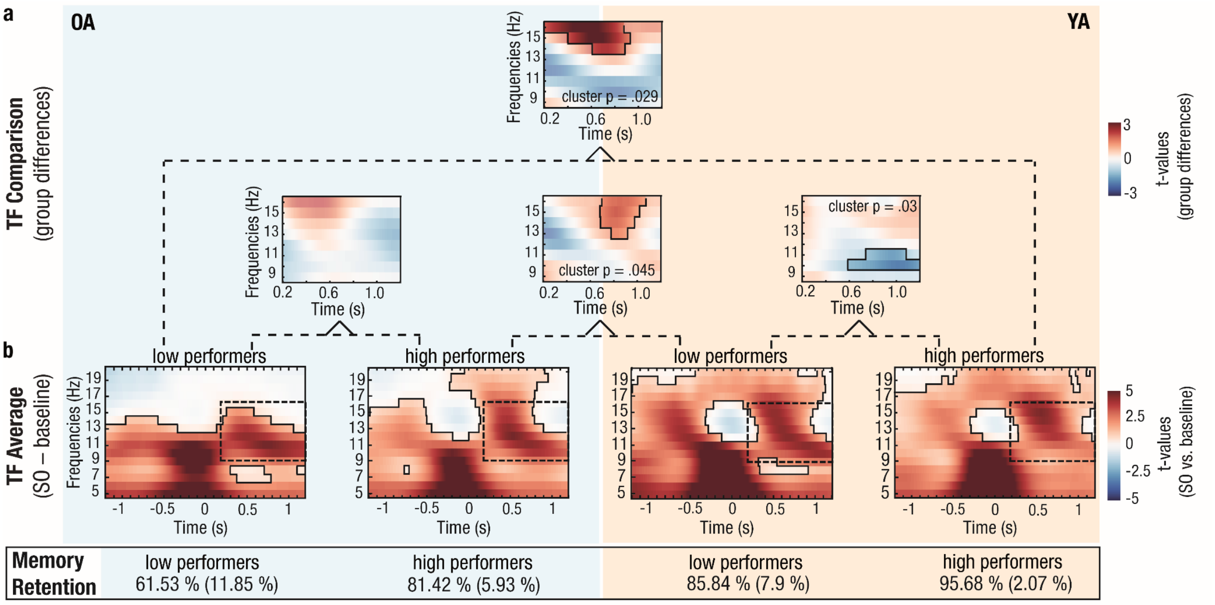

Supplementary Figure S7. Central SO–SP coupling changes as a function of memory retention. Participants of each age group are split into low and high performers based on their ability to retain memories overnight (median split, mean performance and standard deviation for each group are indicated below the time–frequency plots). (b) For each subgroup SO-modulated EEG activity is depicted (differences in wavelet power for SO trials (respective trough ± 1.2 s)) compared to baseline trials (in *t*-score units, reference window for group contrasts outlined by dashed black line). Significant clusters (cluster-based permutation test, cluster $\alpha < 0.05$) are highlighted and outlined. (a) Time–frequency comparison of high and low performers within each age group, high performing older and low performing younger adults, and high performing younger and low performing older adults, respectively. Significant clusters (cluster-based permutation test, cluster $\alpha < 0.05$) are highlighted and outlined. Note the negative cluster in the within age group comparison of younger adults (cluster $p = .03$) congruent with the significant negative correlation within this age group. YA: younger adults; OA: older adults; SO: slow oscillation; SP: spindle; TF: time–frequency.

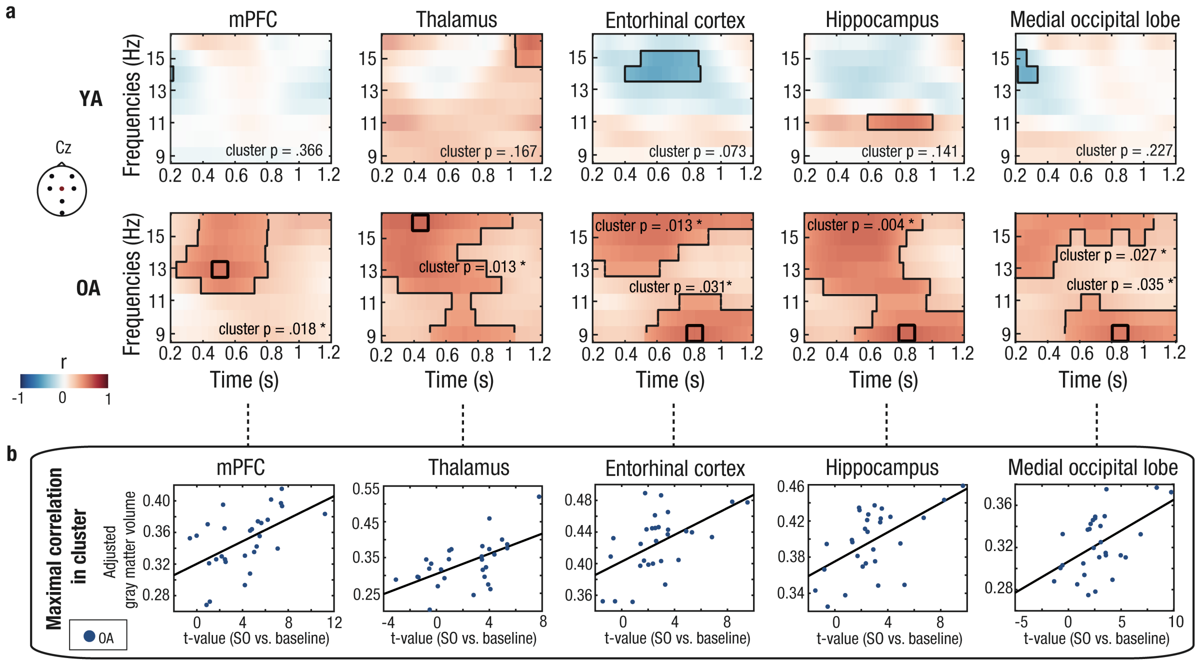

Supplementary Figure S8. Central SO–SP coupling relates to brain integrity in old age. (a) Pearson's correlation coefficients between brain volume in extracted ROIs and neuronal activity during SO up-states are displayed for both younger and older adults. In each plot the cluster with the smallest *p*-value (after control for multiple comparisons using a cluster-corrected correlation approach) is highlighted and outlined and its significance (cluster $\alpha < 0.05$) is marked by asterisks. In older adults, significant positive correlation clusters were detected for mPFC, thalamus, entorhinal cortex, hippocampus, and medial occipital lobe. More positive values in this cluster are associated with greater brain volume in the respective ROI. All extracted ROIs relate to enhanced neuronal activity during the SO up-state. For mPFC and thalamus, source regions of SO and SP generation, the effects are more specific to fast SP frequencies. Greater volume in entorhinal cortex, hippocampus, and medial occipital lobe relates to a broader enhancement of neuronal activity reaching into the slow SP frequency band. No significant correlation cluster was found in younger adults. (b) Maximal positive correlation between SO-related power modulations (*t*-values) and adjusted ROI gray matter volume in the cluster (least-squares fit line shown in black) in older adults. The corresponding time–frequency points are highlighted in the respective clusters in panel (a). Note. Scatter plots only serve illustrative purposes. Hence, no significance is stated in the respective subplots. YA: younger adults; OA: older adults; mPFC: medial prefrontal cortex; ROI: region of interest; SO: slow oscillation; SP: spindle.
